# Fluorodopa is a Promising Fluorine‐19 MRI Probe for Evaluating Striatal Dopaminergic Function in a Rat Model of Parkinson's Disease

**DOI:** 10.1002/jnr.23983

**Published:** 2016-10-28

**Authors:** Daijiro Yanagisawa, Keisuke Oda, Masatoshi Inden, Shigehiro Morikawa, Toshiro Inubushi, Takashi Taniguchi, Masanori Hijioka, Yoshihisa Kitamura, Ikuo Tooyama

**Affiliations:** ^1^ Molecular Neuroscience Research Center Shiga University of Medical Science Seta Tsukinowa‐cho Otsu 520‐2192 Japan; ^2^ Department of Neurobiology Kyoto Pharmaceutical University 5 Nakauchi‐cho Yamashina‐ku Kyoto 607‐8414 Japan; ^3^ Laboratory of Medical Therapeutics and Molecular Therapeutics Gifu Pharmaceutical University 1‐25‐4 Daigaku‐Nishi Gifu 501‐1196 Japan; ^4^ Laboratory of Pharmacology and Neurobiology, College of Pharmaceutical Sciences Ritsumeikan University 1‐1‐1 Noji‐higashi Kusatsu 525‐8577 Japan; ^5^ Present address: Present address: Ono Pharmaceutical Co., Ltd

**Keywords:** fluorodopa, fluorine‐19 MRI, dopaminergic neuron, Parkinson's disease

## Abstract

Parkinson's disease (PD) is a progressive neurodegenerative disorder characterized by the loss of dopaminergic neurons in the substantia nigra projecting to the striatum. It has been estimated that approximately 80% of the striatal dopamine and 50% of nigral dopaminergic neurons are lost before the onset of typical motor symptoms, indicating that early diagnosis of PD using noninvasive imaging is feasible. Fluorine‐19 (^19^F) magnetic resonance imaging (MRI) represents a highly sensitive, easily available, low‐background, and cost‐effective approach to evaluate dopaminergic function using non‐radioactive fluorine‐containing dopaminergic agents. The aim of this study was to find a potent ^19^F MRI probe to evaluate dopaminergic presynaptic function in the striatum. To select candidates for ^19^F MRI probes, we investigated the following eight non‐radioactive fluorine‐containing dopaminergic agents: fluorodopa (F‐DOPA), F‐tyrosine, haloperidol, GBR13069 duhydrochloride, GBR12909 duhydrochloride, 3‐bis‐(4‐fluorophenyl) methoxytropane hydrochloride, flupenthixol, and fenfluramine. In ^19^F nuclear magnetic resonance measurements, F‐tyrosine and F‐DOPA displayed a relatively higher signal‐to‐noise ratio value in brain homogenates than in others. F‐DOPA, but not F‐tyrosine, induced the rotational behavior in a 6‐hydroxydopamine (6‐OHDA)‐induced hemiparkinsonian rat model. In addition, a significantly high amount of F‐DOPA accumulated in the ipsilateral striatum of hemiparkinsonian rats after the injection. We performed ^19^F MRI in PC12 cells and isolated rat brain using a 7T MR scanner. Our findings suggest that F‐DOPA is a promising ^19^F MRI probe for evaluating dopaminergic presynaptic function in the striatum of hemiparkinsonian rats. © 2016 Wiley Periodicals, Inc.

**Abbreviations**
^19^F: Fluorine‐19
PD: Parkinson's disease
MRI: magnetic resonance imaging
F‐DOPA: fluorodopa
6‐OHDA: 6‐hydroxydopamine
PD: Parkinson's disease
DA: dopamine
PET: positron emission tomography
DAT: dopamine transporter
VMAT2: vesicular monoamine transporter 2
NMR: nuclear magnetic resonance
L‐DOPA: levodopa
AADC: aromatic L‐amino acid decarboxylase
FOV: field of view
TR: repetition time
TE: echo time
CSI: chemical shift imaging
HPLC: high‐performance liquid chromatography
DHBA: 3,4‐dihydroxybenzylamine hydrobromide
SEM: standard error of the mean
ANOVA: analysis of variance
BBB: blood brain barrier
DOPAC: dihydroxyphenylacetic acid
MAO: monoamine oxidases
COMT: catechol‐O‐methyltransferase
3‐MT: 3‐methoxytyramine
HVA: homovanillic acid

## INTRODUCTION

Parkinson's disease (PD) is a progressive neurodegenerative disorder characterized by the loss of dopaminergic neurons in the substantia nigra projecting to the striatum. The clinical diagnosis of PD is based on the presence of motor symptoms, including rest tremor, bradykinesia, rigidity, and loss of postural reflexes. However, it has been estimated that approximately 80% of striatal dopamine (DA) and 50% of nigral dopaminergic neurons are already lost before the onset of typical motor symptom [Pavese and Brooks, 2009; Wu et al, 2011], indicating that an early diagnosis of PD using noninvasive imaging is feasible.

Recent advances in imaging techniques such as positron emission tomography (PET) and single photon emission computed tomography enable us to evaluate neural function of the brain in vivo. Imaging of the dopaminergic function in PD was first reported with [^18^F] fluorodopa ([^18^F]‐DOPA) PET and has been further extended in imaging studies of dopamine transporter (DAT) and vesicular monoamine transporter 2 (VMAT2) [Shen et al, 2014; Stoessl et al, 2014]. All these markers demonstrate reduced uptake in the striatum of patients with PD. Thus, these imaging techniques could assist physicians in three critical ways: differential diagnosis of other parkinsonian syndromes; early diagnosis of high‐risk patients (genetic predisposition or environmental exposure to toxin); and monitoring drug treatment [Zhu et al, 2014].

Magnetic resonance imaging (MRI) is another promising method for noninvasive imaging of the brain. Recently, fluorine‐19 (^19^F) MRI techniques with ultra‐high field MR scanner have gained attention based on the following advantages (Higuchi et al., 2005): the MR sensitivity of ^19^F is relatively high compared with that of various nuclei, except ^1^H (^1^H, 100%; ^19^F, 83%; ^31^P, 6.6%; and ^13^C, 1.6%); ^19^F atoms do not exist in biological tissues, thus producing low endogenous background noise; and ^19^F is a non‐radioactive isotope, with 100% natural abundance. Therefore, ^19^F MRI represents a highly sensitive, easily available, low‐background, and cost‐effective approach to evaluate dopaminergic functions using non‐radioactive fluorine‐containing dopaminergic agents.

The aim of this study was to find a potent ^19^F MRI probe for evaluating dopaminergic presynaptic function in the striatum. We investigated eight non‐radioactive fluorine‐containing dopaminergic agents as candidates for ^19^F MRI probes. From the screening, we selected fluorodopa (F‐DOPA) for further investigation and performed neurochemical analysis of F‐DOPA in a 6‐hydroxydopamine (6‐OHDA)‐induced hemiparkinsonian rat model.

## MATERIALS AND METHODS

### Chemicals

The following fluorine‐containing dopaminergic agents were used: F‐DOPA (Advanced Biochemical Compounds, Radeberg, Germany), F‐tyrosine (SynQuest, Alachua, FL, USA), F‐L‐tyrosine (Tokyo Chemical Industry, Tokyo, Japan), haloperidol (a dopamine D2 receptor antagonist; Sigma, St. Louis, MO, USA), GBR13069 dihydrochloride (GBR13069 dihydrochloride; a dopamine transporter inhibitor; Tocris, Bristol, UK), GBR12909 dihydrochloride (GBR12909; a dopamine transporter inhibitor; Tocris), 3‐bis‐(4‐fluorophenyl) methoxytropane hydrochloride (3‐bis‐FPMT; a dopamine transporter inhibitor; Tocris), flupenthixol (a non‐selective dopamine receptor antagonist; Sigma), and fenfluramine (a serotonin releaser; Sigma).

### Cell Culture

The rat adrenal pheochromocytoma cell line PC12 purchased from ATCC (CRL‐1721; Manassas, VA, USA) was cultured in Dulbecco's modified Eagle's medium supplemented with 10% (v/v) fetal bovine serum, 5% horse serum, 100 unit/mL penicillin, and 100 µg/mL streptomycin and kept at 37°C in a humidified incubator with 5% CO_2_/95% air. The sera were inactivated at 56°C for 30 min before use.

Confluent PC12 cells in a 10‐cm culture dish were treated with 5 mM F‐DOPA or 5 mM F‐tyrosine and were incubated for 30 min at 37°C. After washing with PBS three times, the cells were collected. Then, 25 × 10^6^ cells in 400 µL of PBS were subjected to ^19^F nuclear magnetic resonance (NMR) analysis and ^19^F MRI as described below.

### Animals

Twenty male Wistar rats weighing 280 g at 8‐10 weeks of age were used in the present study (Japan SLC; Hamamatsu, Japan). The rats were maintained at 21°C under a 12‐h light/dark cycle (lights on 08:00–20:00 h) in standard laboratory cages with free access to food and water throughout the study period. The rats were acclimated to the environment for at least 1 day prior to the experiment. All experimental procedures in this study were approved by the Committees on Animal Care and Use of Shiga University of Medical Science and Kyoto Pharmaceutical University.

Rats were perfused through the aorta with 150 mL of 10 mM phosphate buffered saline (pH 7.4, PBS) under deep anesthesia induced by intraperitoneal (i.p.) injection of sodium pentobarbital (50 mg/kg). The brains were quickly removed and homogenized in two volumes of PBS, using a homogenizer (Microtec, Funabashi, Japan). The brain homogenate was used for *in vitro* NMR analysis as described below. Alternatively, the isolated brain was injected with 10 µL of 20 µg/µL F‐DOPA by a 25‐µL Hamilton syringe with a 26‐gauge needle after removal. The F‐DOPA‐injected brain was subjected to MRI analysis, as described below.

### Hemiparkinsonian Rat Model

For stereotaxic microinjection, rats were anesthetized (sodium pentobarbital, 50 mg/kg, i.p.) and immobilized in a Kopf stereotaxic frame. Subsequently, 32 nmol 6‐OHDA (Sigma) in a final volume of 4 µL of sterilized physiological saline containing 0.02% ascorbic acid (1.1 mM, as a 6‐OHDA stabilizer) was injected into the left substantia nigra pars compacta of rats (Kitamura et al., 2010). The intranigral injection coordinates of 4.8 mm anterior–posterior, 1.8 mm left lateral, and 7.8 mm ventral from the bregma were derived from a rat brain atlas (Paxinos and Watson, 2005). The injection was delivered by a motor‐driven 10 µL Hamilton syringe using a 26‐gauge needle. The infusion rate was 1 µL/min, and the injection needle was kept in place during 5 min after injection. After the surgery, the rats were kept warm under a heating lamp during anesthesia recovery. No animals were excluded from the study. An assay of rotational behavior was performed twice at 10 and 17 days after the surgery. The MR measurement was performed with an interval of more than 7 days after the assay of rotational behavior.

### Assay of Rotational Behavior

Drug‐induced rotational asymmetry was assessed in rotometer bowls, as described previously (Inden et al., 2006). In brief, the numbers of full body turn rotations in the ipsilateral and contralateral directions were counted for 60 min after the drug injection. The rotational behavior induced by levodopa (L‐DOPA) (10 mg/kg, i.p.; Sigma) was measured 10 days after the operation in rats that were treated with an aromatic L‐amino acid decarboxylase (AADC) inhibitor, benserazide (6.25 mg/kg, i.p.; Sigma), 15 min before L‐DOPA injection. Seven days later, the rotational behavior induced by F‐DOPA (10 mg/kg, i.p.) or F‐L‐tyrosine (10 mg/kg, i.p.) was measured in rats that received benserazide (6.25 mg/kg, i.p.) 15 min before the injection.

### NMR

The NMR spectra were determined using a high performance 270‐MHz NMR spectrometer (JNM‐GX270; JEOL, Tokyo, Japan). Chemical shifts of the ^19^F NMR signals were referenced to C_6_F_6_ at −163 ppm as an external standard (Amatsubo et al., 2009; Yanagisawa et al., 2010).

Fluorine‐containing dopaminergic agents were each dissolved in water at 1 mM and 4 mM concentrations. The 4 mM aqueous solution was mixed with three volumes of rat brain homogenate at a final concentration of 1 mM. The 1 mM aqueous solutions and mixtures were each transferred to a NMR tube (5‐mm diameter) to obtain the ^19^F NMR spectra.

### MRI

We used a 7.0 T horizontal‐bore MR scanner (Unity Inova; Varian, Palo Alto, CA) (Amatsubo et al., 2009; Yanagisawa et al., 2011; Yanagisawa et al., 2014). A home‐built solenoid‐type volume coil measuring 2.5 cm in diameter and 2.8 cm in length and tuned to both ^1^H and ^19^F frequencies (300 MHz and 282 MHz, respectively) was used to collect the data. First, ^1^H spin‐echo MR images of the mouse brain were obtained with a 25 mm × 25 mm field of view (FOV), 2‐mm slice thickness in the axial plane, 256 × 128 resolution, 30‐ms echo time (TE), and 1000‐ms repetition time (TR). A nonlocalized ^19^F NMR spectrum was obtained from the whole head using a single pulse sequence with 8192 data points, 40,000‐Hz SW, 1‐s TR, and 900 acquisitions. The ^19^F chemical shift imaging (CSI) data were collected with 40,000‐Hz SW, 24 mm × 24 mm FOV in the axial plane, and 56 acquisitions for each 8 × 8 phase‐encoding step. A slice‐selective pulse was not used, but slice selection was achieved by the sensitivity of the RF coil. Whole signals covered by the coil sensitivity were acquired.

For ex vivo MRI, the rat brain was removed, and then F‐DOPA (200 µg) was injected into the brain with a microsyringe. After that, ^19^F MRI of ex vivo rat brain was performed for 10 min. For ex vivo ^19^F MRI in the hemiparkinsonian rat brain, F‐DOPA (10 mg/kg, i.p.) was injected in the hemiparkinsonian rats that received benserazide (6.25 mg/kg, i.p.) 15 min before the injection. The rats were sacrificed by decapitation 30 min after the F‐DOPA injection, the brain was quickly removed, and the isolated brain was then placed in the MR scanner.

### High‐performance Liquid Chromatography (HPLC) for F‐DOPA

The HPLC system consisted of a solvent delivery system (Σ871, Irica, Kyoto, Japan), a column oven (CTO‐10AS, Shimaszu, Kyoto, Japan), and an amperometric detector (E‐502, Irica). Chromatographic separation was accomplished using a COSMOSIL cholester packed column 4.6 mm I.D. × 250 mm (Nacalai Tesque, Kyoto, Japan). The column was coupled to a COSMOSIL cholester guard column 4.6 mm I.D. × 100 mm (Nacalai Tesque). An aliquot of the sample was injected onto the column and eluted with a mobile phase containing 3% acetonitrile, 0.68% sodium acetate, 0.065% sodium 1‐heptanesulfonate, and 0.01% EDTA using the isocratic HPLC method. The flow rate was 1.0 mL/min, and the column oven temperature was 40°C.

For sample preparation, 250 µL extraction reagent, 0.2 M perchloric acid, 0.02 M EDTA, and 0.4 µM 3,4‐dihydroxybenzylamine hydrobromide (DHBA; as an internal standard) were added to the cell pellet or rat striatum, and the mixture was homogenized by sonication. The homogenates were centrifuged, and the supernatants were filtered through a 0.2‐µm membrane filter. The amounts of F‐DOPA were directly measured from the aliquots.

### Statistical Analysis

Statistical analyses were performed in GraphPad Prism 5 (GraphPad Software, La Jolla, CA, USA; RRID:SCR_002798). Results are given as the mean ± standard error of the mean (SEM). Statistical significance was determined by unpaired two‐tailed Student's t‐test for single comparisons, and an analysis of variance (ANOVA) followed by Bonferroni test for multiple comparisons. P < 0.05 was considered significant.

## RESULTS

### Screening Test Using ^19^F NMR Analysis

**Figure 1:**
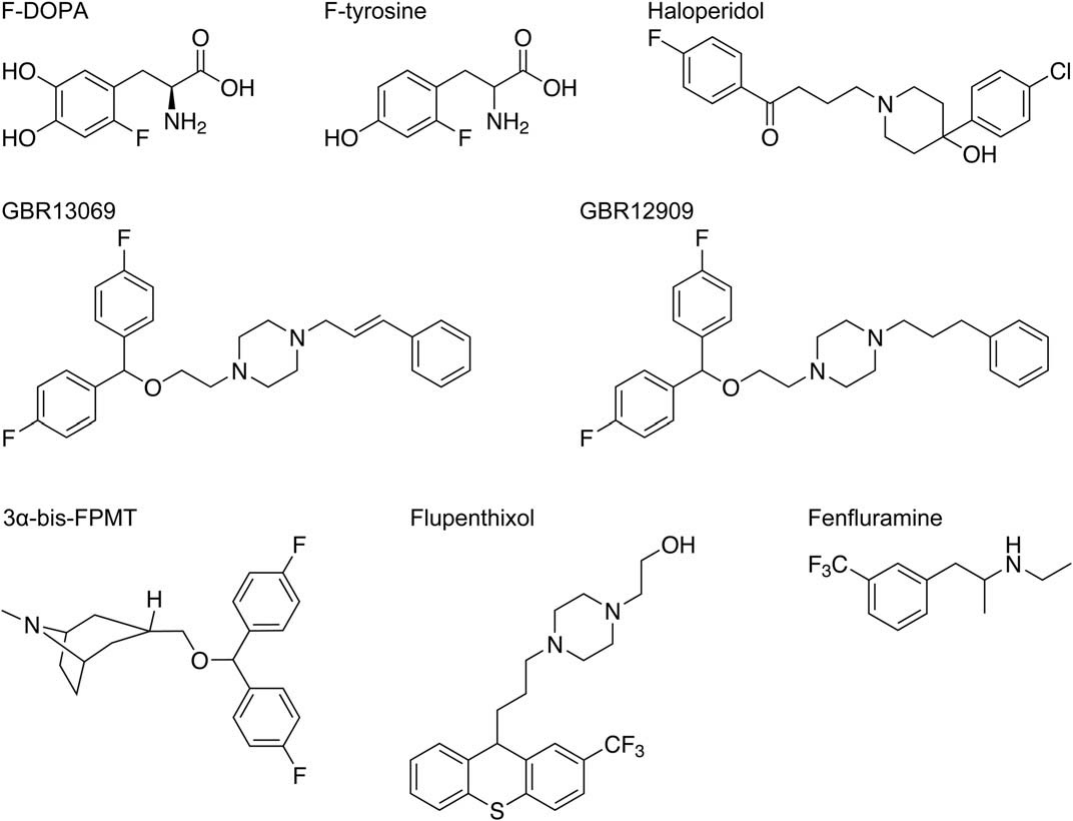
Chemical structures of fluorine‐containing dopaminergic agents used in the present study.

**Figure 2:**
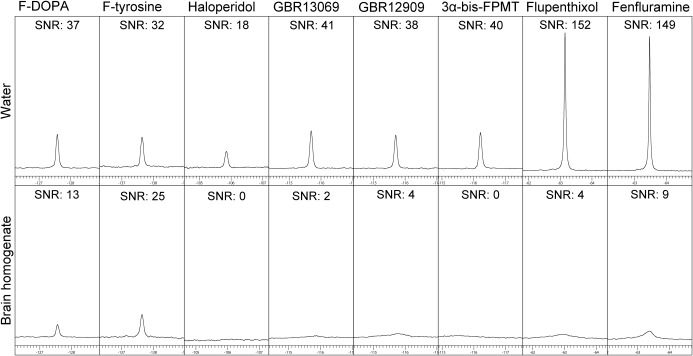
^19^F nuclear magnetic resonance (NMR) spectra in fluorine‐containing dopaminergic agents. Eight compounds were subjected to ^19^F NMR in aqueous solutions (upper) and brain homogenates (lower).

We measured ^19^F NMR spectra of fluorine‐containing dopaminergic agents to identify a candidate ^19^F MRI probe. We screened the following eight compounds: F‐DOPA, F‐tyrosine, haloperidol, GBR13069, GBR12909, 3‐bis‐FPMT, flupenthixol, and fenfluramine (Fig. 1 and Table 1). The ^19^F NMR spectra of these compounds each showed an intense peak and high signal‐to‐noise ratio (S/N) in aqueous solutions (Fig. 2 and Table 1). However, the shape of the peak was broadened and the intensity was dramatically decreased when the compounds were mixed with brain homogenates. Although S/N values for haloperidol, GBR13069, GBR12909, 3‐bis‐FPMT, flupenthixol, and fenfluramine decreased by almost one tenth in brain homogenates, F‐DOPA and F‐tyrosine displayed relatively higher S/N values in brain homogenates (Fig. 2 and Table 1). Therefore, we selected F‐DOPA and F‐tyrosine as candidate ^19^F MRI probes.

**Table 1:** Fluorine‐Containing Dopaminergic Agents used in this Study and the Summary of ^19^F NMR Analysis

Chemical			F‐DOPA	F‐tyrosine	Haloperidol	GBR13069	GBR12909	3a‐bis‐FPMT	Flupenthixol	Fenfluramine
Molecular weight			215.2	199.2	375.9	448.5	450.6	357.4	436.5	231.3
CLogP			0.16	0.55	3.49	5.85	6.03	4.58	4.84	3.46
Fluorine			1	1	1	2	2	2	3	3
^19^F NMR	Water	Chemical shift (ppm)	−127.18	137.63	−105.84	−115.69	−115.71	−116.21	−63.05	−63.47
		SNR	37	32	18	41	36	40	152	149
	Brain homogenate	Chemical shift (ppm)	−127.17	−137.64	Undetectable	−115.87	−115.71	Undetectable	−63.01	−63.38
		SNR	13	25	0	2	4	0	4	9

### Pharmacological Properties of F‐DOPA and F‐tyrosine in Hemiparkinsonian Rats

**Figure 3:**
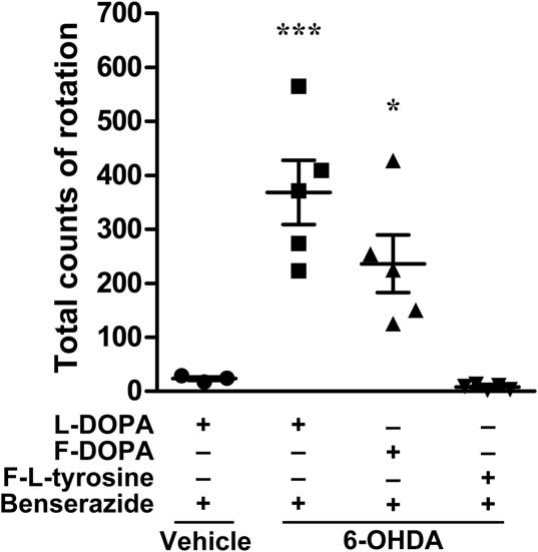
Rotational asymmetry induced by F‐DOPA in hemiparkinsonian rats. The number of rotations was counted for 60 min after drug injection with or without the preinjection of benserazide. Data represent means ± standard error of the mean. Vehicle + L‐DOPA 23.3 ± 3.5 (n = 3); 6‐OHDA + L‐DOPA 368.4 ± 59.31 (n = 5); 6‐OHDA + F‐DOPA 236.2 ± 53.2 (n = 5); 6‐OHDA + F‐DL‐tyrosine 7.6 ± 2.2 (n = 3). Significance (ANOVA followed by Bonferroni test): F(3,14) = 15.22, p = 0.0001 in ANOVA, *p < 0.05, ***p < 0.001 vs. vehicle + L‐DOPA in Bonferroni test.

To investigate the pharmacological properties of F‐DOPA and F‐tyrosine, we analyzed drug‐induced rotational asymmetry in hemiparkinsonian rats. Rotational behavior in the contralateral direction reaching 350 turns for 75 min was induced in hemiparkinsonian rats by the injection of L‐DOPA in combination with benserazide (vehicle + L‐DOPA 23.3 ± 3.5, n = 3 vs. 6‐OHDA + L‐DOPA 368.4 ± 59.31, n = 5; F(3,14) = 15.22, p = 0.0001 in ANOVA; p < 0.001 in Bonferroni test; Fig. 3). Rotational behavior was also induced by the injection of F‐DOPA in combination with benserazide (vehicle + L‐DOPA 23.3 ± 3.5, n = 3 vs. 6‐OHDA + F‐DOPA 236.2 ± 53.2, n = 5; p < 0.05 in Bonferroni test), but not by F‐tyrosine (vehicle + L‐DOPA 23.3 ± 3.5, n = 3 vs. 6‐OHDA + F‐DL‐tyrosine 7.6 ± 2.2, n = 3; p > 0.05 in Bonferroni test).

### ^19^F NMR Analysis and Imaging of Intracellular F‐DOPA in PC12 Cells

**Figure 4:**
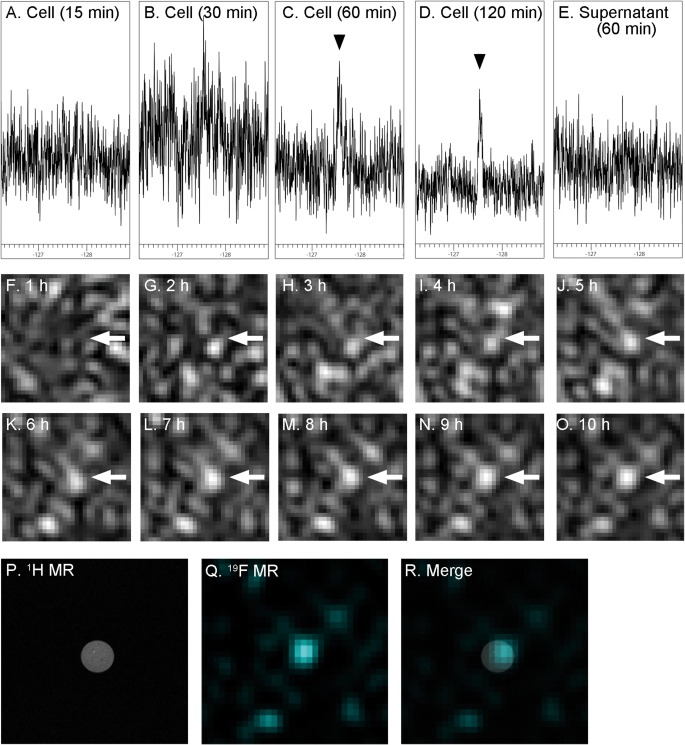
^19^F nuclear magnetic resonance (NMR) analysis and imaging of intracellular F‐DOPA in PC12 cells. (A‐E) ^19^F NMR spectra were measured in the cell suspension of PC12 cells treated with F‐DOPA for 15 (A), 30 (B), 60 (C), and 120 min (D). After that, ^19^F NMR spectrum of the supernatant of the cells was obtained (E). (F‐O) CSI data for ^19^F magnetic resonance imaging (MRI) of intracellular F‐DOPA in the cell suspension were corrected for 1 h, and the measurement were added up to 10 h. (P‐R) Merged image (R) showed that ^19^F MR signal in ^19^F MRI (Q) was colocalized with the position of the cell suspension in ^1^H MRI (P).

Next, we next investigated whether F‐DOPA was taken up by cells and whether the ^19^F NMR signal of intracellular F‐DOPA can be detected in PC12 cells. The ^19^F NMR spectrum of the suspension of PC12 cells treated with F‐DOPA showed an obvious peak, while it took 60 min to detect the obvious peak (Fig. 4A‐D). In contrast, no peak was observed in the ^19^F NMR spectrum of the supernatant, which was obtained by centrifugation of the cell suspension (Fig. 4E).

Next, we performed ^19^F MRI of intracellular F‐DOPA in the cell suspension of F‐DOPA‐treated PC12 cells using a 7T MR scanner. Although several hours were required to detect the F‐DOPA by CSI measurement (Fig. 4F‐O), we found an intense ^19^F MR signal colocalized with the position of the cell suspension in the ^1^H MR image (Fig. 4P–R).

**Figure 5:**
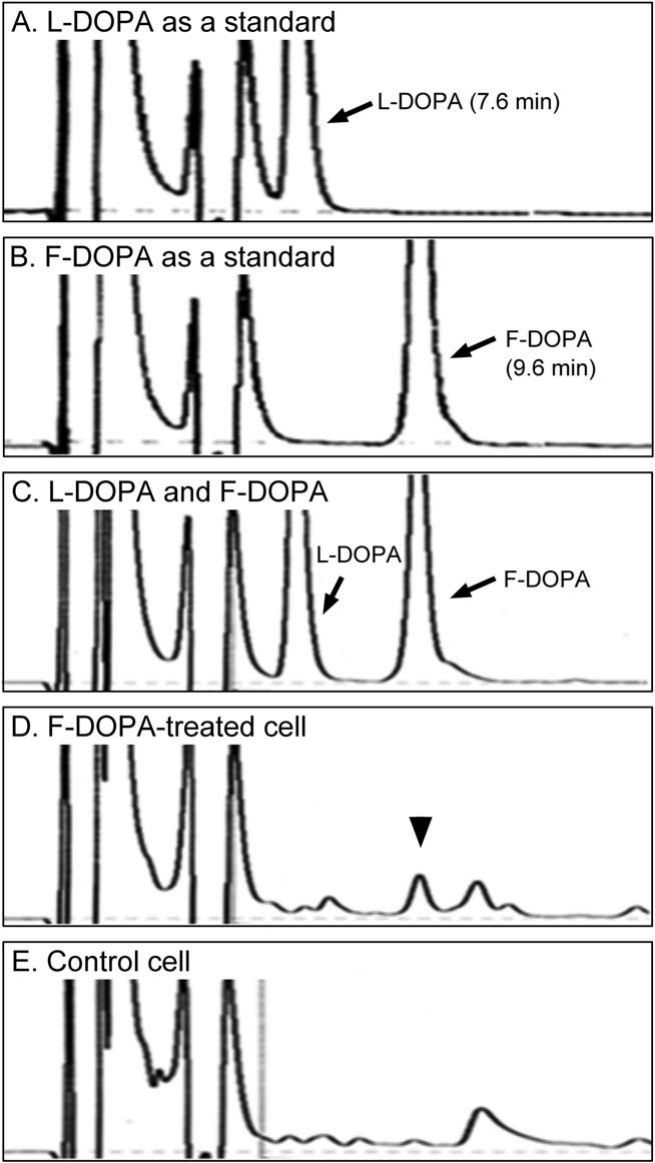
Evaluation of intracellular F‐DOPA content in PC12 cells. Representative HPLC charts showing standard L‐DOPA (A), F‐DOPA (B), and combination of L‐DOPA and F‐DOPA (C) revealed that peaks of L‐DOPA and F‐DOPA were separable. The peak of F‐DOPA was detected in PC12 cells treated with F‐DOPA (D). However no peak was apparent in the cell without F‐DOPA treatment (E).

In HPLC analysis, 24.9 ng of F‐DOPA was detected in 52.1 mg of PC12 cells that were treated with F‐DOPA (Fig. 5). However, no detectable peak was observed in the cell without F‐DOPA treatment. These results suggested that F‐DOPA was taken up by the cells, and ^19^F MR signal in the intracellular F‐DOPA was detectable using a 7T MR scanner.

### ^19^F MRI of *ex vivo* Rat Brain

**Figure 6:**
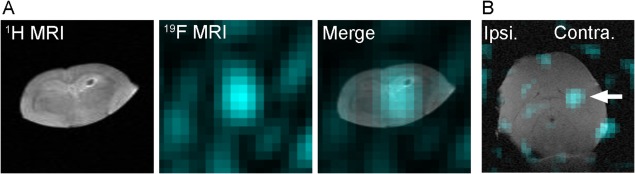
^19^F magnetic resonance imaging (MRI) of ex vivo rat brain. (A) The rat brain was removed, and then F‐DOPA (200 µg) was injected into the brain with a microsyringe. After that, the ^19^F MRI of ex vivo rat brain was performed for 10 min. Chemical shift imaging (CSI) measurement revealed a strong ^19^F MR signal at the injection site. (B) The hemiparkinsonian rats received the intraperitoneal injection with F‐DOPA after the injection with benserazide, and then the brains were removed for ex vivo MRI. ^19^F MRI of ex vivo hemiparkinsonian rat brain in the horizontal plane showed an intense signal (cyan) in the contralateral brain (arrow).

To investigate whether the ^19^F MR signal of F‐DOPA was detected in the ex vivo brain using a 7T MR scanner, we performed ^19^F MRI of ex vivo rat brain that was microinjected with F‐DOPA. The CSI measurement revealed a strong ^19^F MR signal in the injection site of the isolated brain (Fig. 6A). Furthermore, we performed MRI of the ex vivo brain of hemiparkinsonian rats that received the intraperitoneal injection with F‐DOPA. ^19^F MRI in the horizontal plane showed an intense signal in the contralateral side of the ex vivo hemiparkinsonian rat brain by 12‐h measurement (Fig. 6B).

### F‐DOPA Accumulated in the Injured Side of Hemiparkinsonian Rats

**Figure 7:**
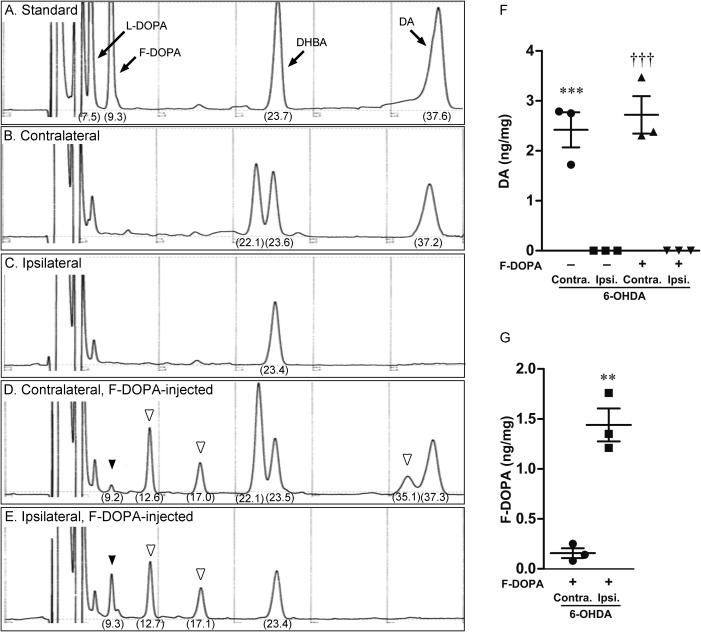
Intraperitoneally injected F‐DOPA accumulated in the striatum of hemiparkinsonian rats. (A) The peaks of L‐DOPA, F‐DOPA, DHBA (internal standard), and DA were detected at 7.5, 9.3, 23.7, and 37.6 min, respectively. (B‐E) To measure DA and F‐DOPA levels, the contralateral (B and D) and ipsilateral (C and E) striata in 6‐OHDA‐induced hemiparkinsonian rats with (D and E) or without (B and C) the injection of F‐DOPA were subjected to HPLC analysis. Black and white arrowheads indicate F‐DOPA and its metabolites, respectively. (F) Loss of DA in the ipsilateral striatum was observed in the hemiparkinsonian rats. Data represent means ± SEM. 6‐OHDA contralateral 2.42 ± 0.35 (n = 3); 6‐OHDA ipsilateral 0 ± 0 (n = 3); 6‐OHDA contralateral + F‐DOPA 2.72 ± 0.38 (n = 3); 6‐OHDA ipsilateral + F‐DOPA 0 ± 0 (n = 3). Significance (ANOVA followed by Bonferroni test): F(3,8) = 33.63, p < 0.0001 in ANOVA, ***p < 0.001 vs. 6‐OHDA ipsilateral, ^†††^p < 0.001 vs. 6‐OHDA ipsilateral + F‐DOPA in Bonferroni test. (G) F‐DOPA‐injected hemiparkinsonian rats showed higher F‐DOPA level in the ipsilateral striatum than the contralateral side. Data represent means ± SEM. 6‐OHDA contralateral + F‐DOPA 0.16 ± 0.05 (n = 3); 6‐OHDA ipsilateral + F‐DOPA 1.44 ± 0.17 (n = 3). Significance (unpaired two‐tailed Student's t‐test): F(2,2) = 10.99, p = 0.1668 in F test, **p < 0.0017 vs. 6‐OHDA contralateral + F‐DOPA in unpaired two‐tailed Student's t‐test.

To test whether F‐DOPA accumulated in the striatum in 6‐OHDA‐induced hemiparkinsonian rats, we injected F‐DOPA (10 mg/kg, i.p.) into benserazide (6.25 mg/kg, i.p.)‐preinjected hemiparkinsonian rats and evaluated the F‐DOPA content in the intact and injured sides of the striatum. In HPLC analysis, the peaks of L‐DOPA, F‐DOPA, DHBA (internal standard), and DA were detected at 7.5, 9.3, 23.7, and 37.6 min, respectively, after mixing with brain homogenates. The peak of DA was undetectable in the ipsilateral striatum of hemiparkinsonian rats, whereas a high peak was detected on the contralateral side (Fig. 7B, C). Additionally, the quantitative analysis showed loss of DA in the ipsilateral striatum (6‐OHDA contralateral 2.42 ± 0.35, n = 3 vs. 6‐OHDA ipsilateral 0 ± 0, n = 3; 6‐OHDA contralateral + F‐DOPA 2.72 ± 0.38, n = 3 vs. 6‐OHDA ipsilateral + F‐DOPA 0 ± 0, n = 3; F(3,8) = 33.63, p < 0.0001 in ANOVA, ***p < 0.001 vs. 6‐OHDA ipsilateral, ^†††^p < 0.001 vs. 6‐OHDA ipsilateral + F‐DOPA in Bonferroni test; Fig. 7F). F‐DOPA‐injected hemiparkinsonian rats showed a small peak of F‐DOPA at 9.2 min and three peaks (probably representing its metabolites) at 12.6, 17.0, and 35.1 min in the contralateral striatum (Fig. 7D). In contrast, there was a higher F‐DOPA peak at 9.3 min and only two peaks at 12.7 and 17.1 min in the ipsilateral striatum (Fig. 7E). Quantitative analysis showed large amounts of F‐DOPA in the ipsilateral striatum of hemiparkinsonian rats in comparison with the intact side (6‐OHDA contralateral + F‐DOPA 0.16 ± 0.05, n = 3 vs. 6‐OHDA ipsilateral + F‐DOPA 1.44 ± 0.17, n = 3; F(2,2) = 10.99, p = 0.1668 in F test, **p < 0.0017 vs. 6‐OHDA contralateral + F‐DOPA in unpaired two‐tailed Student's t‐test; Fig. 7G).

## DISCUSSION

In the present study, we revealed that F‐DOPA is a potent ^19^F MRI probe for evaluating dopaminergic function in the striatum. In ^19^F NMR measurements, F‐tyrosine and F‐DOPA displayed a relatively high peak in the brain homogenates, compared with haloperidol, GBR13069, GBR12909, 3‐bis‐FPMT, flupenthixol, and fenfluramine. F‐DOPA, but not F‐tyrosine, induced rotational behavior in hemiparkinsonian rats, indicating that F‐DOPA crossed the blood‐brain barrier (BBB) and acted like L‐DOPA. In addition, there was a difference in F‐DOPA content in the contralateral and ipsilateral sides of the striatum in hemiparkinsonian rats. Taken together, our findings show that ^19^F MRI of F‐DOPA could be useful for evaluating dopaminergic function in the striatum.

We previously reported that the hydrophilicity or hydrophobicity of the ^19^F MRI probe is a key factor for successful MRI in the brain (Amatsubo et al., 2009). Highly hydrophobic probes may be trapped by the lipid components of the brain such as myelin. This inhibits molecule mobility and causes shortening of T_2_ and broadening of the NMR signal, thereby reducing the NMR signal (Amatsubo et al., 2009). Although high S/N values of ^19^F NMR were detected in aqueous solutions, this was dramatically decreased in brain homogenates. Furthermore, the S/N values were further reduced when the compounds that contain more fluorine atoms were used. This could be because of the high hydrophobicity of fluorine atoms. Our findings demonstrated that F‐DOPA and F‐tyrosine, which have the smallest numbers of fluorine atoms, displayed the highest S/N values in brain homogenates. ^19^F MRI revealed that significantly greater amounts of the injected F‐DOPA accumulated in the ipsilateral striatum of hemiparkinsonian rats than in the contralateral side. Based on these results, we propose that F‐DOPA represents a potent ^19^F MRI probe for evaluating dopaminergic function in the striatum.

The BBB permeability of the compounds should be considered for ^19^F MRI of the brain. In general, high hydrophobicity of a chemical contributes to high BBB permeability. At the same time, the high hydrophobicity of the chemical results in interaction with hydrophobic brain tissues, which leads to a drastic reduction in the NMR signal in the brain, as mentioned above. Therefore, we first performed ^19^F NMR analysis to select the compounds that showed higher S/N signal in brain homogenates. Next, selected compounds were investigated for the pharmacological property and the BBB permeability by rotational behavior in hemiparkinsonian rats. The results showed that low hydrophobic F‐DOPA crossed the BBB, and acted like L‐DOPA. L‐DOPA is transported across the BBB by an amino acid transporter, and thus, it is likely that the amino acid transporter contributes to the BBB permeability of F‐DOPA.

L‐DOPA, a precursor of DA, is one of the most effective drugs in the clinical treatment of PD. L‐DOPA can cross the BBB and is converted to DA by AADC in the brain, which is then stored in intraneuronal vesicles (Arai et al., 1994). Treatment with L‐DOPA and the peripherally acting AADC inhibitor can improve symptoms in PD patients by increasing DA levels in the striatum (Goetz et al., 2005). After acting as a neurotransmitter, DA is converted to 3,4‐dihydroxyphenylacetic acid (DOPAC) and 3‐methoxytyramine (3‐MT) by monoamine oxidases (MAO) and catechol‐O‐methyltransferase (COMT), respectively. DOPAC and 3‐MT are then degraded to form homovanillic acid (HVA) by COMT and MAO, respectively. Interestingly, both L‐DOPA and F‐DOPA induced rotational behavior in the contralateral direction in hemiparkinsonian rats. The contralateral rotational response is a commonly used measure of behavioral sensitization to L‐DOPA and is triggered by the supersensitivity of DA receptors in the DA‐depleted striatum. Thus, F‐DOPA and/or its metabolites (including F‐DA) may be able to stimulate striatal DA receptors in hemiparkinsonian rats. HPLC analysis of F‐DOPA‐injected hemiparkinsonian rats revealed lower F‐DOPA levels in the intact side of the striatum than in the injured side. This suggests that F‐DOPA was readily converted to metabolites such as F‐DA by AADC through the same pathway as L‐DOPA (Cuming et al., 1987; Firnau et al., 1987; Melega et al., 1991; Melega et al., 1990). However, although only two peaks representing possible metabolites were detected in the ipsilateral striatum, an additional peak was detected at a retention time of 35.1 min in the intact striatum. These results implied that the metabolic pathway of F‐DOPA and/or its metabolites was altered in the injured striatum because of presynaptic dopaminergic neurodegeneration. Based on our findings, we suggest that F‐DOPA is readily metabolized in the intact striatum although presynaptic dopaminergic neurodegeneration may alter enzymatic metabolism in the striatum. This induces the accumulation of F‐DOPA and its metabolites (including F‐DA) that can stimulate DA receptors in the ipsilateral striatum. This causes contralateral rotational behavior in hemiparkinsonian rats. Taken together, our findings suggest that the increased F‐DOPA level in the injured striatum of hemiparkinsonian rats reflects presynaptic dopaminergic neurodegeneration. Surprisingly, the ^19^F MR signal was detected in the contralateral side of the ex vivo brain of F‐DOPA‐injected hemiparkinsonian rats, although HPLC analysis showed that the F‐DOPA level in the contralateral striatum was lower than that in the ipsilateral side. The metabolites of F‐DOPA were likely to contribute to ^19^F MR signal in the contralateral side; however, the detailed mechanisms remain unknown and future study in needed to clarify them.

There were some limitations to the application of ^19^F MRI for measuring F‐DOPA *in vivo* in the brain. The most important limitation was the sensitivity of ^19^F MRI. In the present study, it took 5 h to acquire a ^19^F MRI for 24.9 ng of intracellular F‐DOPA in 52.1 mg of PC12 cells. A large amount of injected F‐DOPA accumulated in the ipsilateral striatum of hemiparkinsonian rats; therefore, detecting F‐DOPA *in vivo* by ^19^F MRI would take too long, even with the 7T MR scanner. A number of modifications are required to improve the sensitivity of this method, including the use of an MR scanner with a higher magnetic power, modifying the chemical structure of F‐DOPA, and optimizing the methodology.

We have demonstrated that F‐DOPA may be useful in evaluating dopaminergic function in the striatum. Further studies are needed to improve MRI techniques for the *in vivo* detection of F‐DOPA using ^19^F MR. We believe that investigations into ^19^F MRI of F‐DOPA may provide a novel avenue for PD diagnosis.

## CONFLICTS OF INTEREST

The authors declare that they have no potential conflicts of interest, including financial, personal, or other relationships, which could inappropriately influence or be perceived to influence the work presented here.

## ROLE OF AUTHORS

All authors had full access to all the data in the study and take responsibility for the integrity of the data and the accuracy of the data analysis. Study concept and design: YK, IT Acquisition of data: KO, MI, SM, TI. Analysis and interpretation of data: DY, KO, MI, MH. Drafting of the manuscript: DY, KO. Statistical analysis: DY, KO. Obtained funding: DY, YK, IT. Study supervision: TT, YK, IT.
